# Human embryonic stem cells exert antitumor effects on prostate cancer cells in a co-culture microenvironment

**DOI:** 10.3389/fonc.2023.1164250

**Published:** 2023-05-29

**Authors:** Xinyue Yang, Yang Lu, Qin Kuang, Yong Wu, Xin Tan, Jizhong Lan, Zhe Qiang, Tao Feng

**Affiliations:** ^1^ Key Laboratory of Biochemistry and Molecular Pharmacology of Chongqing, Chongqing Medical University, Chongqing, China; ^2^ College of Pharmacy, Chongqing Medical University, Chongqing, China; ^3^ Chongqing Academy of Chinese Materia Medica, Institute of Pharmacology Toxicology, Chongqing, China

**Keywords:** human embryonic stem cells, prostate cancer, co-culture, antitumor effects, PI3K/Akt/mTOR pathway

## Abstract

Prostate cancer is currently the most common malignancy among men. Given the limitations of current conventional anticancer therapies, new high-risk treatments are urgently needed. Previous studies have shown that embryonic stem cells (ESCs) can reverse the tumorigenic phenotype of tumor cells. However, there are still challenges in using human ESCs (hESCs) directly in cancer treatment. To facilitate the practical application of hESCs, we established a co-culture system consisting of prostate cancer cell lines and hESCs and investigated the antitumor activity of the supernatant of the co-culture system (Co-Sp) *in vitro* and *in vivo*, as well as the underlying mechanisms involved. The Co-Sp decreased the viability of prostate cancer cells in a concentration-dependent manner, significantly inhibited colony formation, and induced cell cycle arrest at the G0/G1 phase of the cell cycle. In addition, Co-Sp promoted apoptosis of prostate cancer cells and inhibited cell migration and invasion. *In vivo* studies also revealed that Co-Sp inhibited tumor growth in the xenograft model. Mechanistic studies showed that Co-Sp reduced the expression of cyclin D1, cyclin E, CDK4, CDK2, MMP-9, MMP-1, and Bcl-2, and increased the expression of p21, cleaved caspase-9, cleaved caspase-3, cleaved PARP, and Bax in prostate cancer cells. Furthermore, the Co-Sp decreased the phosphorylation of PI3K, AKT, and mTOR in cells and tumor tissues. Taken together, our results indicated that the Co-Sp has potent antitumor activity and could directly inhibit tumor growth. Our findings provide a new and effective way for the application of hESCs in cancer therapy and contribute to a new strategy for clinical stem cell therapy.

## 1 Introduction

Prostate cancer is a malignant tumor that threatens the health of men. Due to insidious clinical symptoms in the early stages of prostate cancer, most patients are diagnosed in the advanced stages, when radical surgery is no longer a possible treatment (1). Conversely, the heterogeneous nature of prostate cancer leads to significant individual differences in outcomes of endocrine therapy (2–4). Furthermore, most patients will develop castration-resistant prostate cancer after androgen deprivation therapy and have a high percentage of metastases (3, 5). Multiple chemotherapeutic drugs have been approved to treat advanced prostate cancer, and docetaxel-based chemotherapy has become the standard of first-line therapy (6, 7). However, most chemotherapeutic drugs have strong side effects, as they are extremely cytotoxic, require high doses, and are susceptible to drug resistance (8). Given the limitations of common treatments, there is a need to develop safer and more effective treatments for prostate cancer.

The interaction between cancer cells and the microenvironment promotes the proliferation, metastasis, and tumorigenicity of cancer cells (9–11). Several studies have revealed that the embryonic microenvironment can reverse the malignant phenotype of cancer cells. In fact, the ability of embryonic models to reconvert malignant cells to a normal phenotype has been demonstrated in chick, mouse, and zebrafish embryos (12–15). Tumor cells and embryonic stem cells (ESCs) have similar biological characteristics, such as high self-renewal, infinite proliferation, and signal transduction (16–18). Unlike tumors, ESCs can maintain a microenvironment for self-renewal and differentiation. This difference has led to an increased interest in the role of ESCs in cancer (17). Researchers found that ESCs can play a role similar to that of the early embryonic microenvironment and demonstrated that glioma cell proliferation was significantly inhibited by direct contact with murine ESCs (19). In particular, pretreatment of the microenvironment with hESCs can inhibit the aggressive or tumorigenic phenotype of melanoma cells (20, 21), while the maximal inhibitory effects of human ESCs (hESCs) on melanoma cells can be achieved through direct cell-to-cell contact (22, 23). These findings suggest that the early embryonic microenvironment and ESCs exert strong biological antitumor effects in the context of direct interaction with cancer cells. Therefore, the study of the interactions between ESCs and cancer cells will provide new strategies for cancer therapy.

Although hESCs have great potential for the cellular therapy of diseases, there are risks of teratoma formation and immune rejection after implantation *in vivo*, and thus, presents many challenges and ethical issues for the use of hESCs as a treatment (24–26). Therefore, new therapeutic strategies must be established to overcome these risks while taking full advantage of the significant tumor suppressive activity of hESCs. Based on our previous establishment of a co-culture system consists of hESCs and cancer cells, we hypothesized that supernatant of the co-culture system (Co-Sp) would have antitumor effects. To verify this hypothesis, we established a contact co-culture system of hESCs-prostate cancer cell lines PC3 and DU145, respectively, and collected the Co-Sp to investigate its effects on the activity of tumor cells *in vitro* and *in vivo*.

## 2 Materials and methods

### 2.1 Cell culture and preparation of supernatant

The human prostate cancer cell lines PC3, DU145 and hESCs were purchased from the Cell Bank of the Chinese Academy of Sciences (Shanghai, China). PC3 and DU145 cells were cultured in RPMI 1640 medium (Gibco Invitrogen, Carlsbad, USA) containing 10% FBS (Corning, NY, USA). The hESCs were cultured on Matrigel (Corning, NY, USA) in PSCeasy^®^ II Human Multipotential Stem Cell Culture Medium (Cellapy, Beijing, China). The incubator conditions were 37°C, 5% CO_2_, and 95% air. Before co-culture, PC3 and DU145 cells were cultured with PSCeasy^®^ II Human Multipotential Stem Cell Culture Medium (Cellapy, Beijing, China) for 24 h. When the hESC culture reached 70% confluency, PC3 and DU145 cells at the logarithmic growth stage were added to the hESC culture system at a ratio of 1:2.5 (PC3/DU145 cells: hESCs), respectively. After 72 h of continuous culture, the Co-Sp of PC3-hESCs and DU145-hESCs were collected, respectively. Co-Sp filtered with a 0.22-μm filter membrane, and stored at -80 °C. The supernatant of the individually cultured hESCs (hESC-Sp) was collected using the same method.

### 2.2 CCK8 assay

The Co-Sp and hESC-Sp were diluted with RPMI 1640 to different volume fractions (0%, 20%, 40%, 60%, 80%). Volume fraction (%)=V_supernatant/_V_medium_ × 100%. PC3 and DU145 cells incubated with the supernatant of each concentration group for 48 h, respectively. The absorbance was measured at 450 nm after treating cells with CCK8 reagent (Invitrogen, USA) for 2 h.

### 2.3 Colony formation assay

Co-Sp and hESC-Sp were diluted to the same volume fraction (80%) with RPMI 1640. Cells (500 cells/well) were seeded in a 6-well plate and cultured with the diluted Co-Sp, hESC-Sp, respectively. PC3 and DU145 cells cultured without supernatant were used as negative controls. After 16 days, the colonies were treated with 4% formaldehyde (Beyotime, China) and 0.1% crystal violet dye (Beyotime, China) for 15 minutes each. Finally, the colonies were photographed and counted.

### 2.4 Cell cycle analysis

After cell culture with or without the diluted Co-Sp and hESC-Sp, respectively, PC3 and DU145 cells in each treatment group were resuspended in 100 μL PBS and slowly fixed by adding 900 μL 70% ethanol. Then, 100 μL of RNase A Reagent and 400 μL of propidium iodide (PI) Reagent (50 μg/mL) were added to the cell suspension and thoroughly mixed and incubated at 4°C for 30 min under light protection (Elabscience Biotechnology Co., Ltd, China). Finally, the cell cycle was evaluated using a flow cytometer (BD Biosciences, USA).

### 2.5 Transwell migration and invasion assays

PC3 and DU145 cells (2 × 10^4^ cells/well) were mixed with RPMI 1640 diluted serum-free Co-Sp and hESC-Sp and added to the upper chamber of Transwell plates (Corning, USA) that had been pretreated with Matrigel (Corning, USA) to detect cellular invasion. The lower chamber contained 20% FBS cell culture medium. After 24 h, the chambers were removed, washed twice with PBS, and stained with 0.05% crystal violet for 15 minutes. Cells in the inner walls of the chambers were then gently wiped with a cotton swab. Finally, five randomly selected areas of stained cells were counted and photographed to compare the differences in the number of cells that crossed the Transwell for each group. For the migration assay, Matrigel was not used to coat the Transwell chamber and all other steps were the same as those described above.

### 2.6 Apoptosis assay

After cell treatment, cells were resuspended with pre-chilled PBS at a density of 1 × 10^6^ cells/mL. Subsequently, a suspension of PC3 cells was added, followed by the addition of 5 μL Annexin V-FITC reagent and 5 μL PI Reagent (50 μg/mL) (Biosea, Beijing, China); the culture was incubated for 30 minutes. Finally, apoptosis rates were evaluated using a flow cytometer (BD Biosciences, USA).

### 2.7 Hoechst 33258 staining test

The supernatant of each group was diluted to the same volume fraction (80%) with RPMI 1640. PC3 and DU145 cells were cultured with the diluted supernatant for 48 h and washed twice with PBS. The Hoechst 33258 staining solution (1 mg/mL) (Beyotime, China) was diluted 100 times with PBS to form a working solution and was added to the culture plate to cover the cells and staining was achieved after 15 minutes at room temperature with protection from light. Finally, cells were observed with a light microscope (Nikon, Japan) at 400× magnification.

### 2.8 Quantitative RT-PCR

Total RNA was isolated using the Total Extraction Reagent RNA Isolator (Vazyme Biotech Co., Ltd., China). The cDNA was generated using the HiScript^®^ II 1st Strand cDNA Synthesis Kit (Vazyme Biotech Co., Ltd., China). Gene expression was quantified by RT-PCR (qRT-PCR) using standard PCR kits and SYBR Green qPCR Master Mix (2×) (Bimake, Shanghai, China) using a CFX Manager™ Software sequence detection system (Bio-Rad Laboratories, USA). GAPDH was the reference gene. All data were analyzed according to the 2^-ΔΔCt^ relative quantification method. The primer sequences are listed in **Table 1**.

**Table 1:** Primer sequences used for qRT–PCR.

Primer	Forward	Reverse
GAPDH	CGTATTGGGCGCCTGGTCAC	ATGATGACCCTTTTGGCTCC
cyclin D1	TTCATTTCCAATCCGCCCTCC	TGTGAGGCGGTAGTAGGACAG
cyclin E	GTCCTGGATGTTGACTGCCTTGA	GTCCAGCAAATCCAAGCTGTCTC
CDK2	CTGCTCTCACTGGCATTCCT	TTTCAGGAGCTCGGTACCAC
CDK4	ATGGCTACCTCTCGATATGAGC	CATTGGGGACTCTCACACTCT
Bax	CATGGAGCTGCAGAGGATGAT	TGCTGGCAAAGTAGAAAAGGG
Bcl-2	CGGTGGGGTCATGTGTGTGG	GTGTGCAGGTGCCGGTTCAG
p21	CCTGGTGATGTCCGACCTG	CCATGAGCGCATCGCAATC
MMP1	AAAATTACACGCCAGATTTGCC	GGTGTGACATTACTCCAGAGTTG
MMP9	AGACCTGGGCAGATTCCAAAC	CGGCAAGTCTTCCGAGTAGT

### 2.9 Tumor xenograft assay

Male BALB/c nude mice (4–5 weeks old, 20–30 g) were purchased from Cavens Biogle Model Animal Research Co., Ltd (Jiangsu, China). The animal laboratory was maintained at 25°C ± 1°C, 40–60% humidity, 10–15 air changes per hour, under 10-h light/14-h dark cycles. After the mice were acclimated to the environment for 6 days, PC3 cells (1 × 10^6^) were injected subcutaneously into the mice. When the tumor volume reached approximately 100 mm^3^, mice were randomly selected to receive PBS, Co-Sp, and hESC-Sp treatment. The Co-Sp (200 μL/tumor), hESC-Sp (200 μL/tumor), and PBS (200 μL/tumor) were injected every 48 h at two different sites around the tumor and administered for 21 days. Finally, mice were injected with 2% sodium barbiturate (Westang Co., Ltd., China) for anesthesia and then subjected to a high concentration of carbon monoxide for euthanasia. Tumor tissues were excised, measured, and weighed. All animal experiments were approved by the Ethics Committee of Chongqing Medical University (reference number: 2022042).

### 2.10 Immunohistochemistry

Tumor tissues from different treatment groups were divided into sections. The tissue sections were placed into a citric acid (pH 6.0) antigen retrieval buffer (pH 6.0) (Servicebio, China) for antigen retrieval, and specimens were washed three times with PBS. The sections were sequentially soaked in a 3% hydrogen peroxide solution (Servicebio, China) and 3% BSA (Servicebio, China), each process lasting 25 minutes. Subsequently, specific primary antibodies (anti-Ki67, 1:400, GB111499, Servicebio, China) were added and membranes were incubated at 4°C for 12 h. The sections were immersed in a dilution of HRP-conjugated secondary antibody for 50 minutes. Finally, sections were re-stained with hematoxylin and sealed with sealing gel.

### 2.11 TUNEL assay

Tumor tissues from different treatment groups were frozen in sections. The sections were incubated with a working proteinase K solution for 22 minutes. The sections were rinsed twice with PBS and a permeabilizing working solution (triron stock solution: PBS=1:1000) was added to cover the tissue specimen prior to incubation for 20 minutes. After cleaning the sections, they were first equilibrated in Buffer for 10 minutes and then incubated in a solution containing the TDT enzyme, dUTP (Servicebio, China), and buffer at a ratio of 1:5:50 for 2 h. Finally, the specimens were incubated with DAPI staining solution for 10 minutes.

### 2.12 Western blot analysis

RIPA buffer (Biosharp, USA) was used to lyse cells and tissues, and the protein content was quantified by BCA protein analysis (Beyotime, China). The samples were examined by western blotting. The membranes were incubated with the following specific primary antibodies at 4°C for 12 h: anti-cyclin D1, 1:1000, bs-0623R, Bioss, China; anti-cyclin E, 1:700, WL01072, Wanleibio, China; anti-CDK2, 1:1000, WL01543, Wanleibio, China; anti-CDK4, 1:1500, 11026-1-AP, Proteintech, USA; anti-p21, 1:1000, #2947, Cell Signaling, USA; anti-cleaved caspase-9, 1:1000, WL01838, Wanleibio, China; anti-cleaved caspase-3, 1:1000, WL01992, Wanleibio, China; anti-cleaved PARP, 1:1000, #5625, Cell Signaling, USA; anti-Bax, 1:1000, #AF0120, Affinity, China; anti-Bcl-2, 1:1000, bs-0032R, Bioss, China; anti-MMP9, 1:1000, #AF5228, Affinity, USA; anti-MMP1 1:1000, #AF0209, Affinity, USA; anti-PI3K, 1:1000, #4257, Cell Signaling, USA; anti-AKT, 1:1000, #4691, Cell Signaling, USA; anti-mTOR, 1:1000, #2983, Cell Signaling, USA; anti-p-PI3K, 1:1000, #4228, Cell Signaling, USA; anti-p-AKT, 1:1000, #4060, Cell Signaling, USA; anti-p-mTOR, 1:1000, #5536, Cell Signaling, USA, and β-actin (Bioss, China). Subsequently, the secondary antibody was added and incubated for 2 h. Finally, the bands were imaged by a chemiluminescence immunoassay kit (Zen Bioscience, China) and a luminescence image analyzer (Clinx Scientific Instruments, China).

### 2.13 Statistical analysis

Data are presented as mean ± standard deviation (SD). Significant differences between groups were analyzed using Kruskal–Wallis ANOVA performed using GraphPad Prism V. 5.1.0 (GraphPad Software, Inc., USA). A *P-*value <0.05 was considered statistically significant.

## 3 Results

### 3.1 Co-Sp inhibited the proliferation of prostate cancer cells

**Figure 1:**
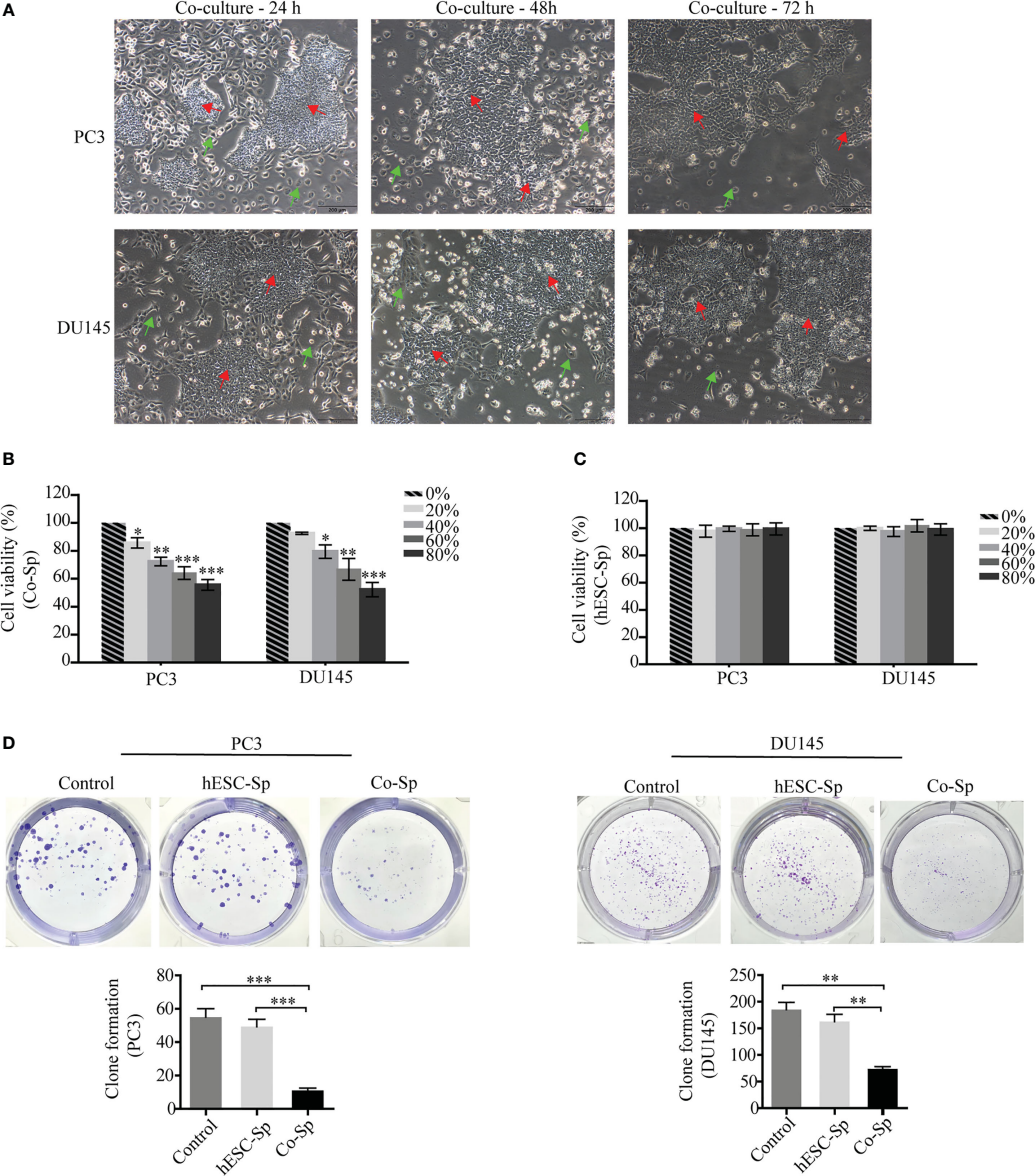
Co-Sp inhibited proliferation of prostate cancer cells. **(A)** hESCs and PC3/DU145 cells co-cultured after 24 h, 48 h, and 72 h observed by microscopy (red arrows: hESCs; green arrows: PC3/DU145 cells. Scale bar, 200 μm). **(B)** The CCK-8 assay indicated that the proliferation rate of PC3 and DU145 cells gradually decreased with increasing Co-Sp concentration. Data indicate mean ± SEM (n=4 biological repeats). *P<0.05, **P<0.01, ***P<0.001, 20%, 40%, 60%, 80% Co-Sp group vs. 0% Co-Sp group. **(C)** The CCK-8 assay showed that the proliferation of PC3 and DU145 cells was not significantly affected by different concentrations of hESC-Sp. Data indicate mean ± SEM (n=4 biological repeats). Not significant, 20%, 40%, 60%, 80% hESC-Sp group vs. 0% hESC-Sp group. **(D)** The plate cloning assay showed that colony formation of PC3 and DU145 cells was reduced after 16 days of Co-Sp treatment. Data indicate mean ± SEM (n=3 biological repeats). **P<0.01, ***P<0.001 vs. the control group and the hESC-Sp group.

After co-culture of PC3 and DU145 cells with hESCs, we found that the proliferation of PC3 and DU145 cells was inhibited, with the number of cells decreasing over time. In particular, the cancer cells near the hESCs were inhibited more significantly. At 72 h, almost no cancer cells were observed around the hESCs (**Figure 1A**). These results indicated that the growth of PC3 and DU145 cells was inhibited in the co-culture system. To investigate whether hESCs secrete factors that influence the proliferation of prostate cancer cells in the co-culture system, we removed the Co-Sp and used it to culture prostate cancer cells alone. Furthermore, we extracted hESC-Sp to serve as a control to exclude the effect of cultures from hESCs cultured individually. The results of the CCK8 assay showed that the cell survival rate of PC3 and DU145 cells gradually decreased with increasing concentrations of Co-Sp (20%, 40%, 60%, and 80%) (**Figure 1B**). However, under the same conditions, there was no significant difference in the survival rate of PC3 and DU145 cells in the different concentration groups (*P*>0.05) (**Figure 1C**). In addition, we performed a colony formation assay using the same concentrations of Co-Sp and hESC-Sp. After 16 days, the number of colonies formed in the Co-Sp group was significantly lower than those formed in the control group (PC3, *P*=0.0005; DU145, *P*=0.0015) and the hESC-Sp group (PC3, *P*=0.0010; DU145, *P*=0.0033) (**Figure 1D**), indicating that the Co-Sp could inhibit the *in vitro* clonogenicity of PC3 and DU145 cells. In short, our results indicated that Co-Sp could inhibit the proliferation of prostate cancer cells.

### 3.2 Exposure to Co-Sp arrested the cell cycle at G0/G1 phase in prostate cancer cells

**Figure 2:**
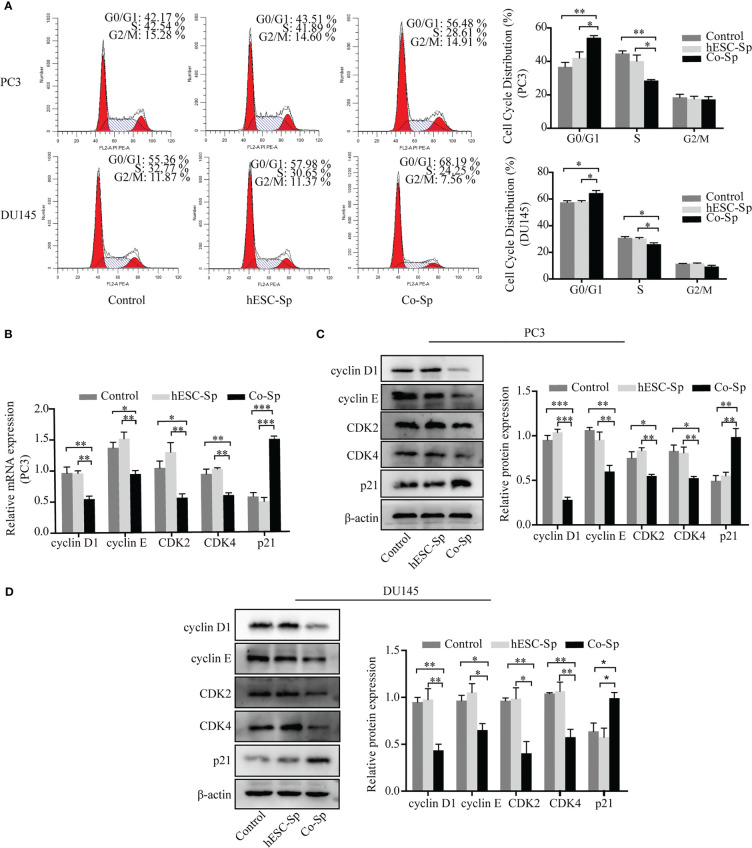
Exposure to Co-Sp arrested the cell cycle at the G0/G1 phase in prostate cancer cells. **(A)** Flow cytometry showed that the proportion of PC3 and DU145 cells in the G0/G1 phase increased and the proportion of cells in the S phase decreased in the Co-Sp group. Data indicate mean ± SEM (n=3 biological repeats). *P<0.05, **P<0.01 vs. control group and hESC-Sp group. **(B)** The expression of cyclin D1, cyclin E, CDK2, CDK4, and p21 in PC3 cells was measured by qRT-PCR. Data indicate mean ± SEM (n=3 biological repeats). *P<0.05, **P<0.01, ***P<0.001 in the Co-Sp-treated vs. control group and hESC-Sp group. **(C, D)** The expression of cyclin D1, cyclin E, CDK2, CDK4, and p21 in PC3 and DU145 cells was measured by western blotting. Data indicate mean ± SEM (n=3 biological repeats). *P<0.05, **P<0.01, ***P<0.001 in the Co-Sp-treated vs. the control group and the hESC-Sp group.

As exposure to Co-Sp was shown to inhibit PC3 and DU145 cell proliferation, we next analyzed whether cell cycle progression was influenced. PC3 and DU145 cells were cultured medium containing different concentrations of supernatant diluted with medium for 48 h and the cell cycle distribution was examined by flow cytometry. After Co-Sp treatment, the proportion of cells in the G0/G1 phase was higher (PC3, 56.48%; DU145, 68.19%) than that in the control group (PC3, 42.17%, *P*=0.0044; DU145, 55.36%, *P*=0.0366) or that in the hESC-Sp group (PC3, 43.51%, *P*=0.0348; DU145, 57.98%, *P*=0.0368) (**Figure 2A**). Next, to explore the mechanism by which the Co-Sp induced G0/G1 cell cycle phase arrest, we analyzed changes in cell cycle regulators known to be involved in the transition from the G0/G1 to S phase, including cyclin D1, cyclin E, CDK4 and CDK2, and p21. The results showed that exposure to Co-Sp negatively regulated the expression of cyclin D1, cyclin E, CDK4, and CDK2, and positively regulated the expression of p21 (**Figures 2B–D**). These results indicated that Co-Sp could arrest the prostate cancer cell cycle in G0/G1 by regulating cell cycle factors.

### 3.3 Co-Sp promoted the apoptosis of prostate cancer cells

**Figure 3:**
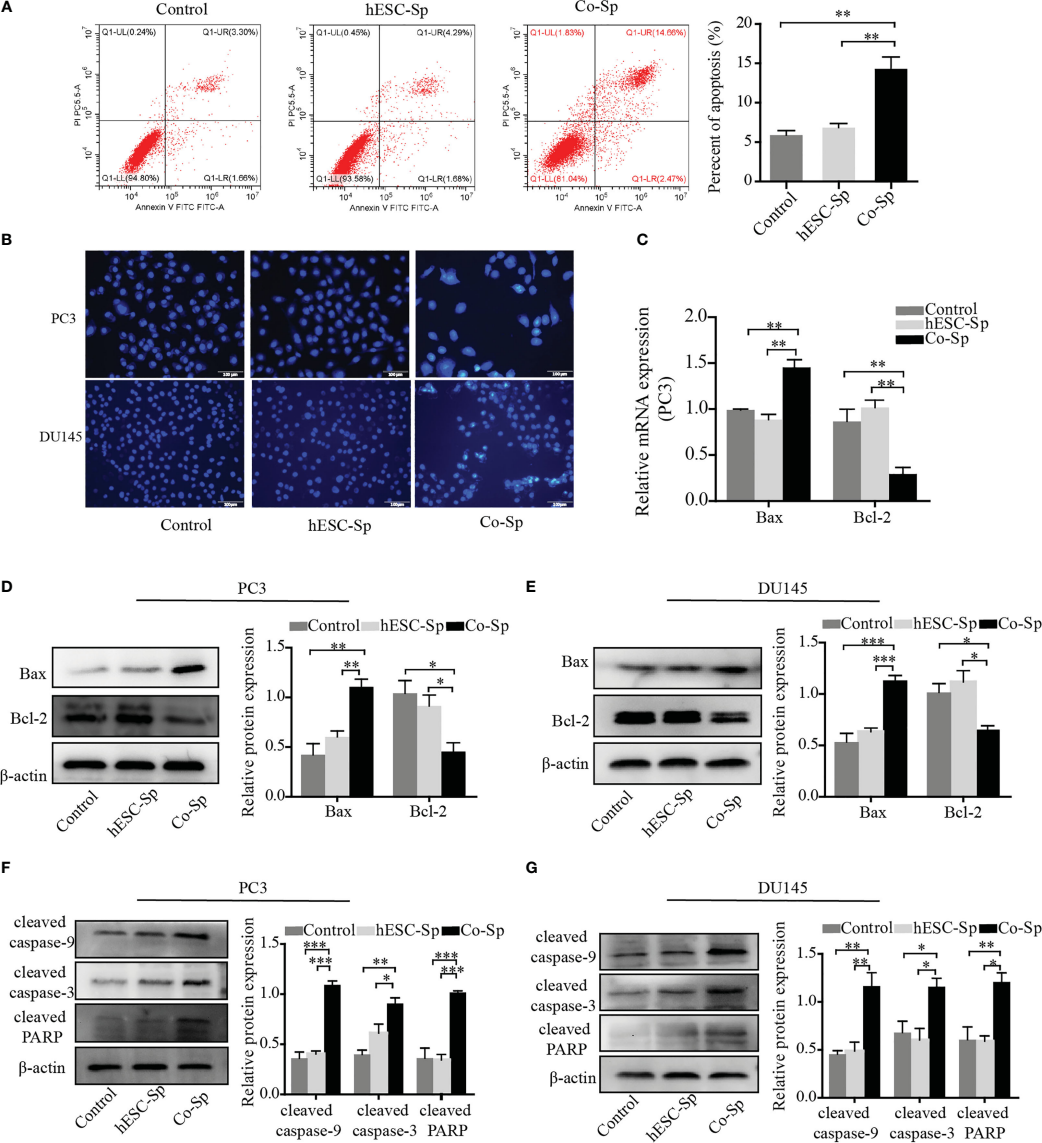
Co-Sp promoted the apoptosis of prostate cancer cells. **(A)** Flow cytometry showed that the PC3 cell apoptosis rate increased after Co-Sp treatment for 48 **(h)** Data indicate mean ± SEM (n=4 biological repeats). **P<0.01 vs. control group and hESC-Sp group. **(B)** The apoptotic morphology of PC3 and DU145 cells stained with Hoechst 33258 was observed under a fluorescence microscope and the apoptotic morphology (nuclear whitening, chromatin condensation, and membrane fragmentation) treated with Co-Sp was observed. **(C)** The expression of Bax and Bcl-2 in PC3 cells was measured by qRT-PCR. Data indicate mean ± SEM (n=3 biological repeats). **P<0.01, in the Co-Sp treated group versus the control group and the hESC-Sp group. **(D, E)** The expression of Bax and Bcl-2 in PC3 and DU145 cells was measured by western blotting. Data indicate mean ± SEM (n=3 biological repeats). *P<0.05, **P<0.01, ***P<0.001 in the Co-Sp-treated vs. control group and hESC-Sp group. **(F, G)** The expression of cleaved caspase-9, cleaved caspase-3, and cleaved PARP in PC3 and DU145 cells was measured by western blotting. Data indicate mean ± SEM (n=3 biological repeats). *P<0.05, **P<0.01, ***P<0.001 in the Co-Sp-treated vs. the control group and the hESC-Sp group.

To examine whether Co-Sp promotes apoptosis in prostate cancer cells, we analyzed PC3 cell apoptosis using the AnnexinV-PI double-staining assay. Flow cytometry revealed that Co-Sp increased the rate of apoptosis of PC3 cells (17.13%) compared to the control group (4.96%, P=0.0063) and the hESC-Sp group (5.97%, *P*=0.0093) (**Figure 3A**). Subsequently, we further performed Hoechst staining on PC3 and DU145 cells. We observed that both PC3 and DU145 cells showed typical apoptotic features after Co-Sp treatment, such as nuclear whitening, chromatin condensation, or nuclear fragmentation, while no morphological changes associated with apoptosis were observed in the control groups (**Figure 3B**). Furthermore, to explore the mechanism of Co-Sp-induced apoptosis, we detected the expression of apoptosis-related factors, including Bax, Bcl2, cleaved caspase-9, cleaved caspase-3, and cleaved PARP. Compared to the control groups, Co-Sp increased Bax expression and decreased Bcl-2 expression (**Figures 3C–E**). Furthermore, the expression levels of cleaved caspase-9, cleaved caspase-3, and cleaved PARP were significantly increased in PC3 and DU145 cells after Co-Sp treatment (**Figures 3F, G**). These results suggested that Co-Sp promoted apoptosis of prostate cancer cells.

### 3.4 Co-Sp attenuated the migration and invasion capability of prostate cancer cells

**Figure 4:**
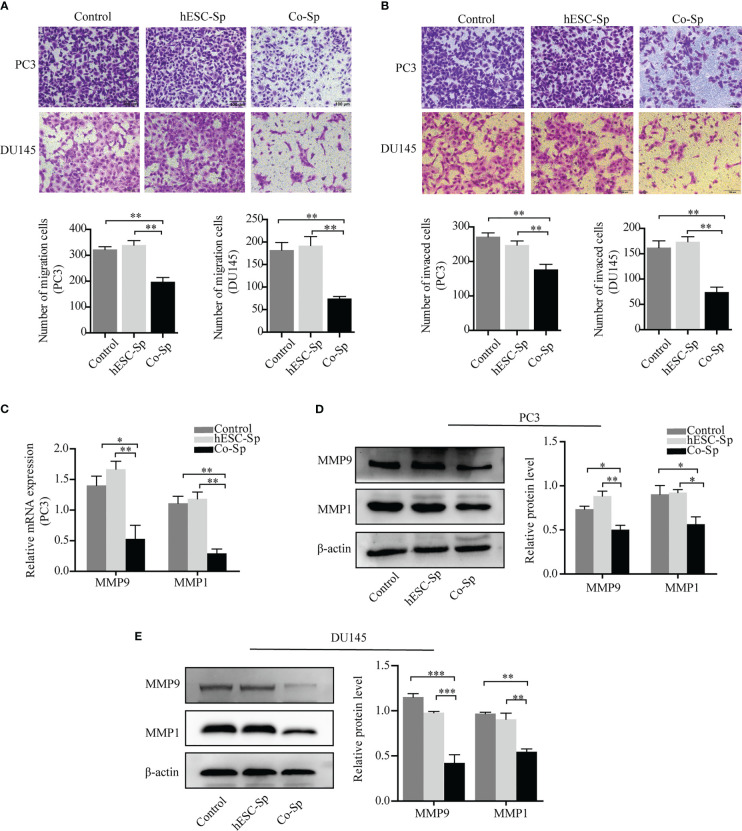
Co-Sp attenuated the migration and invasion ability of prostate cancer cells. **(A)** Transwell migration assay showing that Co-Sp decreased across the membrane of PC3 and DU145 cells. Scale bar, 100 μm. Data indicate mean ± SEM (n=5 biological repeats). **P<0.01 vs. control group and hESC-Sp group. **(B)** Transwell invasion assay showing that Co-Sp reduced the invasion of PC3 and DU145 cells in the membrane. Scale bar, 100 μm. Data indicate mean ± SEM (n=5 biological repeats). **P<0.01 vs. control group and hESC-Sp group. **(C)** The expression of MMP1 and MMP9 in PC3 was evaluated by qRT-PCR. Data indicate mean ± SEM (n=3 biological repeats). *P<0.05, **P<0.01, in the Co-Sp-treated vs. the control group and hESC-Sp group. **(D, E)** The expression of MMP1 and MMP9 in PC3 and DU145 was assessed by western blotting. Data indicate mean ± SEM (n=3 biological repeats). *P<0.05, **P<0.01, ***P<0.001 in the Co-Sp-treated vs. the control group and the hESC-Sp group.

To explore the effects of exposure to the Co-Sp on the migration and invasion of prostate cancer cells, we chose a suitable concentration of Co-Sp (which had less impact on the viability of PC3 and DU145 cells) and used the same concentration of hESC-Sp as a control. First, the Transwell migration assay was performed to evaluate the inhibitory activity of Co-Sp on cell migration. As shown in **Figure 4A**, Co-Sp significantly reduced the number of PC3 and DU145 cells that crossed the membrane compared to the control group (PC3, *P*=0.0031; DU145, *P*=0.0034) and the hESC-Sp group (PC3, *P*=0.0039; DU145, *P*=0.0046). Furthermore, we conducted a Transwell invasion assay to explore the effects of Co-Sp on the invasion ability of PC3 and DU145 cells. The Transwell invasion assay showed that Co-Sp reduced the number of cells that invaded the membrane compared to the control group (PC3, *P*=0.0070; DU145, *P*=0.0059) and the hESC-Sp group (PC3, *P*=0.0084; DU145, *P*=0.0020) (**Figure 4B**). Subsequently, to further explore the mechanisms involved in the inhibitory effects on cell migration and invasion by Co-Sp, we examined key proteolytic enzymes of the matrix metalloproteinase (MMP) family, MMP9 and MMP1. Co-Sp reduced the expression of MMP9 and MMP1 in PC3 and DU145 cells (**Figures 4C–E**). Altogether, our data supported the idea that Co-Sp inhibited the migration and invasion of prostate cancer cells.

### 3.5 Co-Sp inhibited tumor growth in the xenograft model

**Figure 5:**
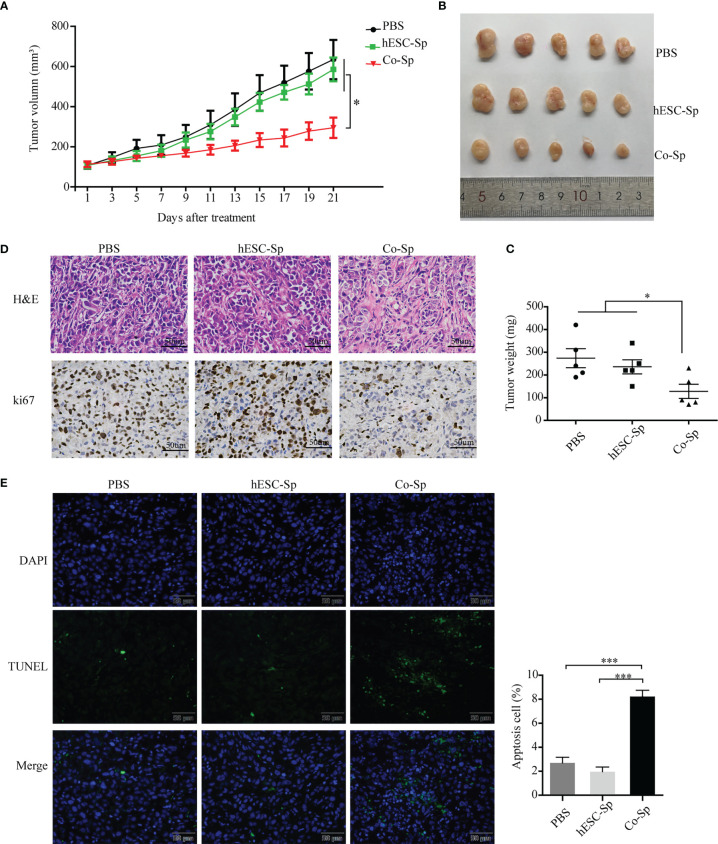
Co-Sp inhibited tumor growth in the xenograft model. **(A)** Changes in tumor volume after Co-Sp, hESC-Sp, and PBS treatment. Data indicate mean ± SEM (n=5 mice per group). *P<0.05, in the Co-Sp group vs. PBS group and hESC-Sp group. **(B)** Images of mouse tumors after 21 days of treatment with Co-Sp, hESC-Sp, and PBS. **(C)** Comparison of tumor weight between Co-Sp, hESC-Sp, and PBS treatment groups. Data indicate mean ± SEM (n=5 mice per group). *P<0.05, in the Co-Sp group vs. PBS group and hESC-Sp group. **(D)** Tumor tissues were detected by hematoxylin and eosin staining, and Ki-67 immunostaining. Scale bar, 100 μm. **(E)** TUNEL staining analysis of tumor tissue apoptosis rate, blue: DAPI; green: TUNEL-positive cells. Scale bar, 50 μm. Data indicate mean ± SEM (n=5 biological repeats). *P<0.05, ***P<0.001, in the Co-Sp group vs. the PBS group and the hESC-Sp group.

To evaluate whether Co-Sp could inhibit tumor growth *in vivo*, we injected PC3 cells subcutaneously into BALB/c nude mice to establish xenograft tumor models. When the tumor volume reached approximately 100 cm^3^, mice were treated by injecting Co-Sp, hESC-Sp, or PBS at two different peritumoral sites, with each injection 48 h apart, for 21 days. During treatment, tumor growth was reduced in mice treated with Co-Sp compared to those treated with PBS or hESC-Sp (**Figure 5A**). As shown in **Figures 5B, C**, the tumors in the Co-Sp-treated mice were smaller than those in the control groups on day 21 (PBS, P=0.023; hESC-Sp, P=0.038), while there were no significant differences between the hESC-Sp-treated group and the PBS-treated group (P>0.05). Ki67 is an indicator of tumor cell proliferation. We found that Ki67 expression was decreased in tumors of Co-Sp-treated mice (**Figure 5D**). Furthermore, the TUNEL assay showed that the ratio of TUNEL+ tumor cells after Co-Sp treatment increased to 9.87% compared to those of the PBS group (1.26%, *P*<0.001) and of the hESC-Sp group (2.83%, *P*<0.001) (**Figure 5E**), indicating that Co-Sp could promote tumor cell apoptosis *in vivo*. In short, these results suggested that the Co-Sp could inhibit the growth of xenograft tumors.

### 3.6 Co-Sp inhibited the PI3K/Akt/mTOR pathway *in vitro* and *in vivo*

**Figure 6:**
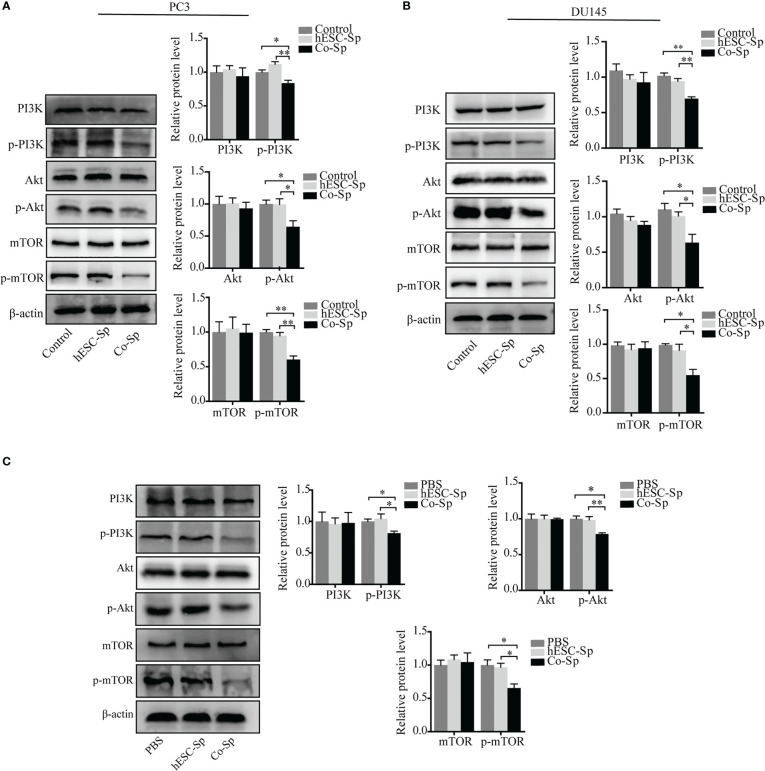
Co-Sp inhibited the PI3K/Akt/mTOR pathway *in vitro* and *in vivo*. **(A, B)** Western blotting analysis showed that Co-Sp down-regulated the expression of phosphorylated (p)-PI3K, p-Akt, and p-mTOR in PC3 and DU145 cells. Data indicate mean ± SEM (n=3 biological repeats). *P<0.05, **P<0.01 vs. control group and hESC-Sp group. **(C)** Western blotting analysis showed that Co-Sp down-regulated the expression of phosphorylated (p)-PI3K, p-Akt, and p-mTOR in tumor tissues. Data indicate mean ± SEM (n=3 biological repeats). *P<0.05, **P<0.01, in the Co-Sp treated vs. the PBS group and in the hESC-Sp group.

The PI3K/Akt/mTOR pathway is involved in the occurrence and development of various tumors (27, 28). To investigate whether the antitumor activities of Co-Sp mentioned above were related to this pathway, we detected the expression of proteins related to PI3K/Akt/mTOR signaling and their phosphorylation status in cells and tissues by western blotting assays. The phosphorylation of PI3K, AKT, and mTOR were decreased in PC3 and DU145 cells following Co-Sp treatment (**Figures 6A, B**). Furthermore, consistent results were observed in the xenograft model. After Co-Sp treatment, PI3K, AKT, and mTOR phosphorylation was down-regulated in tumor tissues (**Figure 6C**). These results indicated that Co-Sp could inhibit the PI3K/AKT/mTOR pathway *in vitro* and *in vivo*.

## 4 Discussion

The great potential of ESCs in biomedical applications lies in their ability to maintain developmental stability and differentiate into various tissues and cells (29–31). With the further exploration of ESCs, several studies have found that the malignant phenotype of tumor cells is reversed in the ESC microenvironment (17, 19). In contrast, differentiated ESCs can contribute to the malignant phenotype of tumor cells (32). Current evidence suggests that the reversal of tumor cells into a benign phenotype by the ESC microenvironment requires cell-cell contact (22, 23). Consistent with these studies, we found that PC3 and DU145 cell growth was inhibited when co-cultured with hESC. However, it is unclear whether secretory factors also play a role. Furthermore, we found that Co-Sp significantly inhibited PC3 and DU145 cell proliferation and promoted apoptosis, while hESC-Sp did not have a tumor suppressor effect, indicating that hESC secreted related factors following their stimulation by cancer cells, which in turn exerted antitumor effects. Furthermore, the Co-Sp blocked cell migration and invasion. *In vivo* studies also revealed that the Co-Sp inhibited tumor growth in the xenograft model. These results suggest that the antitumor effects produced by hESCs in the co-culture system may be achieved through changes in the secreted factors of hESCs.

Cell cycle dysregulation is one of the main reasons for the uncontrolled proliferation of tumor cells (33, 34). To explore the mechanism by which Co-Sp inhibits prostate cancer cell proliferation, we examined whether Co-Sp could block cell cycle progression. The results indicated that Co-Sp increased the proportion of cells in the G0/G1 phase. Cell cycle-related molecules interact with each other to form a complex network responsible for cycle regulation. Two cell cycle kinase complexes, CDK2-cyclin E and CDK4-cyclin D1, are vital regulators of G1-S phase transition (34). p21 negatively regulates the cell cycle and functions by binding to multiple CDK-cyclin complexes to inhibit their activity (35). Consequently, our results showed that exposure to the Co-Sp increased p21 expression and decreased that of cyclin D1, cyclin E, CDK4, and CDK2, indicating that factors present in the Co-Sp could inhibit cell proliferation by regulating the cell cycle progression of PC3 and DU145 cells. Furthermore, regulation of cell cycle progression is closely related to cell reprogramming (36). ESCs have been reported to inhibit tumor growth by promoting reprogramming-induced cell cycle arrest (23). Our study has suggested that the reprogramming effect of hESCs on cancer cells is achieved by altering secretory factors in Co-Sp, which is expected to replace the hESCs for therapeutic use.

Induction of tumor cell apoptosis is an effective means to treat tumors (37). Bax and Bcl-2 are members of the Bcl-2 family, which synergistically regulate mitochondrial function to produce apoptotic effects (38). Furthermore, caspase-9 is the upstream molecule of the mitochondrial apoptotic pathway and can activate downstream apoptotic effector enzymes, leading to the initiation of the caspase cascade reaction (39). Activation of caspase-3 can further cleave different substrates and eventually lead to cell death (40, 41). Consistent with these studies, our results showed that the Co-Sp increased the ratio of Bax/Bcl-2 expression and activated the expression of cleaved caspase-9, cleaved caspase-3, and cleaved PARP in PC3 and DU145 cells, which explains the pro-apoptotic effect of Co-Sp. In addition, we found that Co-Sp attenuated the migration and invasion capability of PC3 and DU145 cells. As members of the MMP family, MMP9 and MMP1 are overexpressed in many tumors with metastatic capacity, including androgen-independent prostate cancer (42, 43). The ESC microenvironment has been reported to inhibit the migration and invasion of A2058 cutaneous melanoma cells by inhibiting the activities of MMP9 and MMP1 (22). Similarly, our results showed that Co-Sp down-regulated the expression of MMP1 and MMP9 in PC3 and DU145 cells, which accounted for the inhibitory effect of Co-Sp on cell migration and invasion. These results suggest that Co-Sp may contain factors that inhibit tumor cell survival and metastasis and has a good potential in the treatment of metastatic tumors.

Finally, we further explored the molecular pathways related to the effects of Co-Sp mentioned above. Several studies have confirmed that activation of the PI3K/AKT/mTOR pathway plays a vital role in the occurrence and development of prostate cancer (44–46). Therefore, we explored the effects of Co-Sp on the PI3K/AKT/mTOR pathway. Our experiments showed that Co-Sp down-regulated the levels of phosphorylation of PI3K, Akt, and mTOR in PC3 and DU145 cells. Furthermore, we examined tumor tissues of mice treated with Co-Sp *in vivo* and found that phosphorylated levels of PI3K, Akt, and mTOR were lower in tissues of Co-Sp-treated tumors. PI3K activation is known to trigger multiple downstream pathways, including Akt activation of G1/S checkpoint control by regulating cycle regulators such as cyclin D1 and CDK4 (47). Akt mediates cell survival through regulation of the Bcl-2-associated death promoter and the caspase-binding protein (42). Additionally, PI3K improves MMP activity by regulating downstream molecules to promote tumor metastasis (48). Therefore, as shown in the present study, the Co-Sp may induce cell cycle arrest, promote cell apoptosis, and block cell metastasis in PC3 and DU145 cells, at least in part due to the suppression of the PI3K/AKT/mTOR pathway.

In conclusion, this study first demonstrated that Co-Sp exerted potent antitumor activity by inhibiting proliferation, blocking migration, and invasion, promoting *in vitro* apoptosis of prostate cancer cells, and inhibiting tumor growth *in vivo*. This inhibitory effect may be partly attributed to the suppression of the PI3K/Akt/mTOR pathway. These findings provide a novel strategy for promoting the application of hESCs in cancer therapy. The use of supernatant from co-culture systems instead of cell therapy is expected to avoid the risks and ethical restrictions associated with hESCs. In addition, the co-culture system of hESCs and cancer cells provides a novel approach for personalized treatment of cancer patients. Nevertheless, due to the complex mechanisms involved in the interactions between hESCs and cancer cells, further investigation is needed to better understand the activity of the antitumor effectors elicited by this co-culture system.

## Data availability statement

The raw data supporting the conclusions of this article will be made available by the authors, without undue reservation.

## Ethics statement

All animal experiments were approved by the Ethics Committee of Chongqing Medical University (reference number: 2022042).

## Author contributions

XY designed the study, conducted the experiment, collected the data, and wrote the manuscript. YL analyzed and interpreted the data. QK and YW conducted the investigation. JL and XT assisted in data collection and manuscript preparation. ZQ and TF conducted project management/supervision and reviewed the manuscript. All authors contributed to the article and approved the submitted version.
